# Adequate prediction for inhibitor affinity of Aβ_40_ protofibril using the linear interaction energy method†

**DOI:** 10.1039/c9ra01177c

**Published:** 2019-04-23

**Authors:** Son Tung Ngo, Binh Khanh Mai, Philippe Derreumaux, Van V. Vu

**Affiliations:** Laboratory of Theoretical and Computational Biophysics, Ton Duc Thang University Ho Chi Minh City Vietnam ngosontung@tdtu.edu.vn; Faculty of Applied Sciences, Ton Duc Thang University Ho Chi Minh City Vietnam; Institute for Computational Science and Technology (ICST), Quang Trung Software City Ho Chi Minh City Vietnam binh.khanh.mai@su.se; Laboratoire de Biochimie Théorique, UPR 9080 CNRS, IBPC, Université Paris Diderot 13 rue Pierre et Marie Curie 75005 Paris France; Laboratory of Theoretical Chemistry, Ton Duc Thang University Ho Chi Minh City Vietnam; Faculty of Pharmacy, Ton Duc Thang University Ho Chi Minh City Vietnam; NTT Hi-Tech Institute, Nguyen Tat Thanh University Ho Chi Minh City Vietnam

## Abstract

The search for efficient inhibitors targeting Aβ oligomers and fibrils is an important issue in Alzheimer's disease treatment. As a consequence, an accurate and computationally cheap approach to estimate the binding affinity for many ligands interacting with Aβ peptides is very important. Here, the calculated binding free energies of 30 ligands interacting with 12Aβ_11–40_ peptides using the linear interaction energy (LIE) approach are found to be in good correlation with experimental data (*R* = 0.79). The binding affinities of these complexes are also calculated by using free energy perturbation (FEP) and molecular mechanic/Poisson–Boltzmann surface area (MM/PBSA) methods. The time-consuming FEP method provides results with similar correlation (*R* = 0.72), whereas MM/PBSA calculations show very low correlation with experimental data (*R* = 0.27). In all complexes, van der Waals interactions contribute much more than electrostatic interactions. The LIE model, which is much less time-consuming than both the FEP and MM/PBSA methods, opens the door to accurate and rapid affinity prediction of ligands with Aβ peptides and the design of new ligands.

## Abstract 2

The efficient approach to estimate inhibitors targeting Aβ oligomers and fibrils is an important issue in Alzheimer's disease treatment.

## Graphical abstract

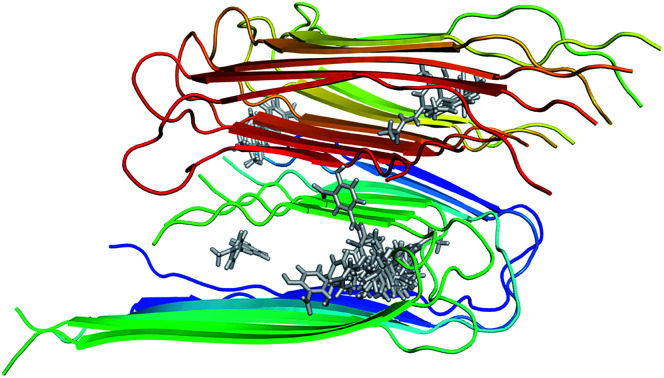

## Introduction

Alzheimer's disease (AD) is a common neurodegenerative disorder in the senior population and has strong negative affection on intellectual abilities.^1–3^ More than 44 million people worldwide suffer from AD leading to a social welfare burden.^4^ Despite extensive biophysical and clinical studies, all drugs targeting Aβ oligomers and amyloid plaques made primarily of the 40 (Aβ_40_) and 42 (Aβ_42_) amino acids have failed and there is currently no efficient treatment against AD.^5–10^ The mechanism of Aβ peptide accumulation in the extracellular space with aging and the memory impairment by Aβ aggregation are still poorly understood. Based on the cascade amyloid hypothesis that Aβ oligomers are the most toxic species,^11^ finding efficient compounds that inhibit or retard the formation of Aβ oligomers and fibrils is a common strategy, for instance natural compounds,^12^ metal chelators,^13^ chaperones,^14^ RNA aptamers,^15^ and short peptides.^16,17^

Computer simulations at different levels of protein representations are an efficient tool to provide deeper insights into the aggregation of Aβ peptides.^18^ Another goal of computational studies is to accurately estimate the binding affinity of protein–ligand complexes facilitating drug development.^19^ In this context, several methodologies have been developed, including free energy perturbation (FEP),^20,21^ thermodynamic integration (TI),^22,23^ molecular mechanic/Poisson–Boltzmann surface area (MM/PBSA),^24–26^ linear interaction energy (LIE),^27–30^ and docking approaches.^31,32^ Because of the large number of ligands to be tested, one have to consider both the accuracy of the calculated binding affinities and the computational costs. Docking methods use simple scoring functions to estimate binding affinities of a large ligand library with low CPU costs. However, because of the lack of flexibility of ligands and proteins and the low accuracy of scoring functions, docking often gives poor results compared with experimental data.^33,34^ On the other hand, FEP/TI method, which calculates absolute binding free energies based on conformational sampling from extensive molecular dynamics (MD) simulations, can provide accurate results, but extremely expensive computational costs have hindered its application to large-scale screening. The end-point MM/PBSA approach, which combines molecular mechanics and continuum solvents to calculate binding free energies, has been successfully applied for several systems.^35–38^ However, the results of this method strongly depend on several factors, such as continuum-solvation method, dielectric constant, charge model, and entropic calculations.^39–41^ The MM/PBSA method is also time consuming for calculating the vibrational entropy of large systems using normal mode analysis. Last but not least, the end-point free energy calculation method, LIE, has also been successfully applied to various systems.^42–49^ In this method, the binding free energy is calculated based on the average van der Waals and electrostatic interaction differences of ligand with its surrounding environments upon association, *i.e.* the free ligand in solvent (free state – denoted as subscript f) and the ligand in complex with solvated protein (bound state – denoted as subscript b).

1 Δ*G*_LIE_ = *α*(〈*V*^vdW^_l–s_〉_b_ − 〈*V*^vdW^_l–s_〉_f_) + *β*(〈*V*^elec^_l–s_〉_b_ − 〈*V*^elec^_l–s_〉_f_) + *γ*

The coefficients *α* and *β* are the scaling factors for nonpolar and polar terms, whereas *γ* is a constant term, which correlates to the change of the hydrophobic nature of the binding site according to different types of ligands.^50^ In some modified versions, the effects of entropic contributions,^42^ solvent-accessible surface area (SASA),^43,51^ intramolecular energies,^52^ and structural descriptors of ligands have also been considered.^47^ Moreover, continuum solvent models have been used rather than explicit solvent models in the original LIE method to reduce computational costs.^53–56^ The performances of the LIE and MM/PBSA methods have been compared in several reports. Their accuracies strongly depend on the systems,^25,46,57–59^ and LIE is less time consuming than MM/PBSA because it does not require any entropy calculations.^41,60^

Computer-aided drug design, which focuses on developing new inhibitors for Aβ peptides and elaborates an efficient method for absolute binding free energy calculations for Aβ peptide systems, has attracted a lot of attention.^61–65^ While in some studies MM/PBSA method provided reasonable results in ranking ligand affinity, the absolute values somewhat did not correlate well with the experimental results.^66–69^ This discrepancy may result from the choice of the dielectric constant and/or the calculation of entropy, which is usually considered for only the last MD-generated structure.^39–41^ To the best of our knowledge, LIE has not been used to calculate binding free energy for Aβ peptide systems. This could be due to the lack of binding poses for Aβ oligomers leading to the difficulty in finding a general parameter set for LIE model. In this work we have calculated the absolute binding free energies of 30 inhibitors interacting with a 12Aβ_11–40_ oligomer using molecular dynamics (MD) simulations and LIE, and compared the results with experimental data and the binding affinities calculated by using the FEP and MM/PBSA methods.

## Materials and methods

### 12Aβ oligomer complexes preparations

**Fig. 1:**
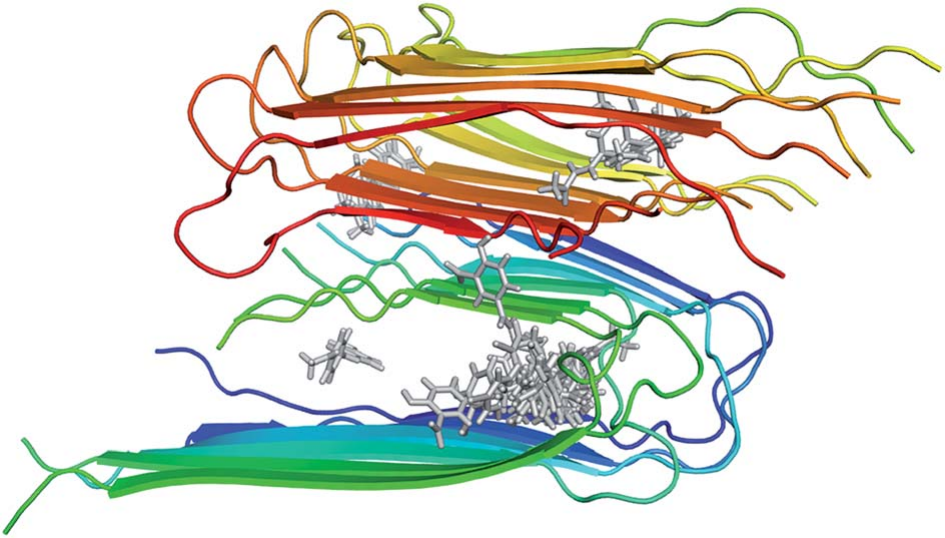
Initial structures used for MD simulations of the 30 inhibitors interacting with the 12Aβ_11–40_ system.

The structure of 12Aβ_11–40_ oligomer was taken from the protein data base (PDB ID: 2LMN) based on numerous constraints from solid state NMR and electron microscopy.^70^ Available inhibitors for Aβ_40_ peptide were searched referring the binding database.^71^ The 3D structures of 30 inhibitors were downloaded from PubChem database (see Table S1 in the ESI† for the list of inhibitors). The geometrics of these compounds was optimized using chemical quantum calculation with B3LYP functional at 6-31G(d) level. The molecular docking protocol using Autodock Vina^72^ was then applied to obtain the Aβ fibril complexes as starting structures for MD simulations. The binding poses of these inhibitors to the 12Aβ_11–40_ oligomer are depicted in Fig. 1.

### Molecular dynamics (MD) simulations

The GROMACS version 5.1.3 (ref. 73) was employed to simulate the solvated complexes. The Amber99SB-ILDN force field^74^ was used to parameterize the protein and counter ions. The general Amber force field^75^ was used to represent the ligands with the help of Ambertools17, in which the atomic charges were obtained using RESP method^76^ with quantum chemical calculations at B3LYP/6-311G(d,p) level. The ligand topologies were then converted to GROMAS format using ACPYPE package.^77^ Finally, the complexes were inserted into the dodecahedron periodic boundary condition water boxes using TIP3P water model.^78^ The smallest distance between a complex to the boundary of water box was chosen as 1.4 nm. The box vectors are approximately (10.4 : 10.4 : 10.4) nm. Therefore, the soluble complex consisted of 12 Aβ_11–40_ peptides, 1 ligand molecule, approximately 24 290 water molecules, and about 12 Na^+^ ions depending on the charge of the ligand.

The soluble complexes were first minimized using the steepest descent scheme. The minimized configurations were then relaxed in NVT and NPT ensembles with 100 ps MD length per simulations. The complexes were restrained by NVT simulations using a small harmonic force and free of restraints by NPT MD simulations. The relaxed system was then used as initial conformation of MD simulations during 20 ns. A total of four MD simulations for each complex were carried out with the same starting structure and different initial velocities. The MD parameters are described in previous studies,^79,80^ in which the non-bonded van der Waals cut-off is 1.0 nm and the PME method is used for electrostatic interactions.

### Data analysis and inhibitor affinity calculation

The root-mean-square-deviations (RMSDs) of Cα atoms were calculated with respect to the initial configuration. A hydrogen bond (HB) is defined based on geometric criterion: distance between acceptor A and donor D is less than 3.5 Å and the A–H–D angle is >135°. A non-bonded contact (NBC) is defined if the distance between any hydrophobic atom, *i.e.* C or S atom, of the ligand and any other atom of Aβ peptides is in range of 2.9–3.9 Å. Diagrams for HB and NBC networks are carried out using LigPlot+ program.^81^ The van der Waals and electrostatic interactions were calculated to build prediction models for LIE method (eqn (1)) with linear regression analysis. Free binding energy of inhibitors with 12Aβ_11–40_ oligomer using the LIE model (eqn (1)) were also compared to MM/PBSA and FEP approaches, successfully applied to several Aβ peptides complexes.^64–66^ Computational details for MM/PBSA and FEP calculations are extensively described in our previous papers.^82,83^

## Results and discussion

### Structures and energies of complexes during MD simulations

The time-evolution of Cα RMSD values of the peptides in the 30 complexes is shown in Fig. S1 in ESI.† The RMSD values increased rapidly at the beginning of simulations and all complexes reached their equilibrium states after 5 ns. In most of cases, the RMSD values are smaller than 0.8 nm, which clearly indicates that these systems are quite stable during the last 15 ns.

Averaged van der Waals and electrostatic interactions between each ligand and Aβ_11–40_ oligomer (bound state) or water (free state) as well as the experimental binding affinity of each ligand are listed in Table S2.† Full references and experimental *K*_i_ values for these inhibitors are provided in ESI.† Except for Thioflavin T (Pubchem ID 16954), the magnitude of electrostatic energies of all other ligands in the bound state are smaller than in the free state, indicating that the 12Aβ_11–40_ oligomer mainly interacts with the ligands by van der Waals interactions. To obtain deeper insights into the binding modes of each inhibitor, 2-dimension protein–ligand interaction diagrams using the last MD-generated structures are drawn in Fig. S2.† All diagrams show that strong van der Waals interactions in all complexes, and a small number of average hydrogen bonds in the 4 MD simulations (Fig. S3†).

### Optimization of LIE equations

We aimed at building a LIE model that can exactly predict binding free energies. Based on previous theoretical studies on the complex of P450_CAM_,^50^ potassium channel,^84^ and aspartic proteases;^85^ the parameters *α* was set to be 0.18, and the parameter *β* was set in the range of 0.33–0.50.^52,86^ Using standard values (*α* = 0.18 and *β* = 0.5), no correlation (*R* ≈ −0.57) was found between the calculated and experimental binding free energies. This is probably due to the lack of a specific binding site for Aβ oligomers, and/or, as mentioned above, the loss of electrostatic interaction of ligand from the free to bound states, which is not observed in other protein complexes.^42–49^

**Fig. 2:**
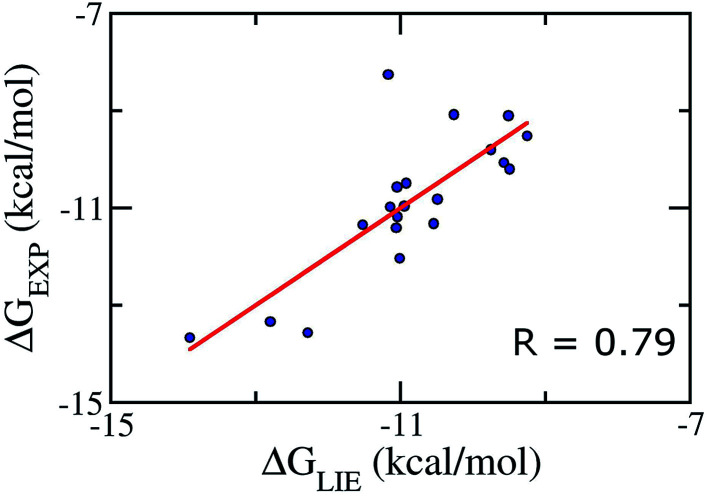
Correlation between experimental binding free energies and that calculated using LIE model (eqn (2)) of training set comprising of 20 complexes.

Because of the failure of these standard *α* and *β* parameters, a new set of parameters for Aβ peptides is needed. Based on the average interaction energies of training set comprising of 20 inhibitors taken randomly listed in Table S4,† the *α*, *β*, and *γ* parameters are calculated to have the values of 0.288, −0.049, and −5.880 kcal mol^−1^, respectively (eqn (2)) giving a Pearson correlation *R* and a standard error of 0.79 and of 0.95 kcal mol^−1^, respectively (Fig. 2).

2 Δ*G*_LIE_ = 0.288(〈*V*^vdW^_l–s_〉_b_ − 〈*V*^vdW^_l–s_〉_f_) − 0.049(〈*V*^elec^_l–s_〉_b_ − 〈*V*^elec^_l–s_〉_f_) − 5.880

In addition, Åqvist and co-workers proposed that for a very small or negative *β* value, the electrostatic interactions in water and in complex with protein can be scaled differently, giving thus different *β*_b_ (for bound state) and *β*_f_ (for free state) values.^52^ Although very small or even negative values of *β* have been found in some studies,^46,87,88^ a small *β* value of −0.049 found in our calculations is still somewhat unexpected. This may result from either the loss of electrostatic interactions of ligands upon association (see Table S2†) or the effect of functional groups of ligands.^89^ Observed results are in good consistent with previous studies that the Aβ inhibitors favorably form domination of vdW interaction energy.^90,91^ It also is in good consistent with the obtained 2-D protein–ligand interaction diagrams (Fig. S2†) where the vdW interaction was found to dominate over the electrostatic interaction.

In addition, the obtained *γ* value was calculated of −5.880 kcal mol^−1^. The large negative value of *γ* indicates small hydrophobic desolvation effects of ligands and very strong hydrophobic interactions between the ligands and 12Aβ_11–40_ oligomer, leading to the deformation of Aβ peptides structures.^50,56^ This is consistent with the fact the van der Waals interactions are dominant in the interaction of ligands and 12Aβ_11–40_ oligomer (see Table S2 and additional data of ESI for details†). The difference is probably caused by the erroneousness of imitating the interaction among constituent molecules, including protein, ligand, and solvated molecules.^92,93^

### Validate the approach

**Fig. 3:**
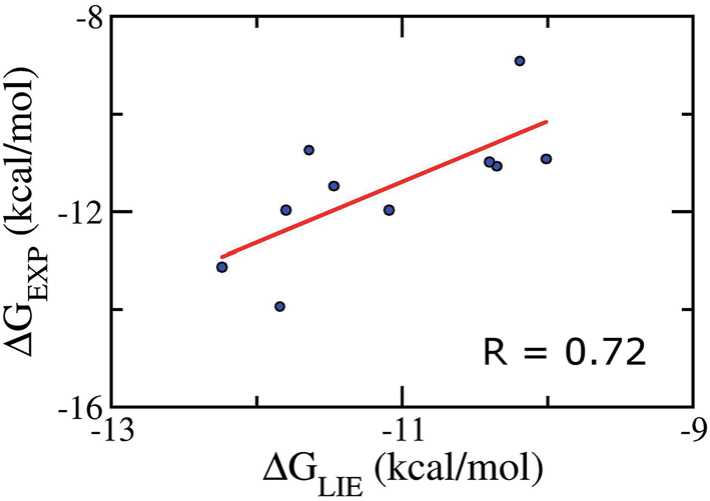
Correlation between experimental binding free energies and that calculated using LIE model (eqn (2)) of testing set comprising of 10 complexes taken randomly listed in Table S5.†

We emphasize that our *α*, *β*, and *γ* parameters and our Pearson correlations do not change if we apply on the test set comprising of 10 inhibitors taken randomly (Table S5†), verifying therefore the correlative power of our model and indicating that our parameter set for the LIE model is rather robust. In particular, the Pearson correlation and standard error were obtained as the values of 0.72 and 1.09 kcal mol^−1^, respectively (Fig. 3). Absolutely, the model accuracy is appropriate to estimate the binding affinity of trial inhibitor to Aβ_40_ system. Whereas, the small error means that is able to categorize ligands revealing similar binding affinities.

### Comparing LIE model with MM/PBSA and FEP methods

**Fig. 4:**
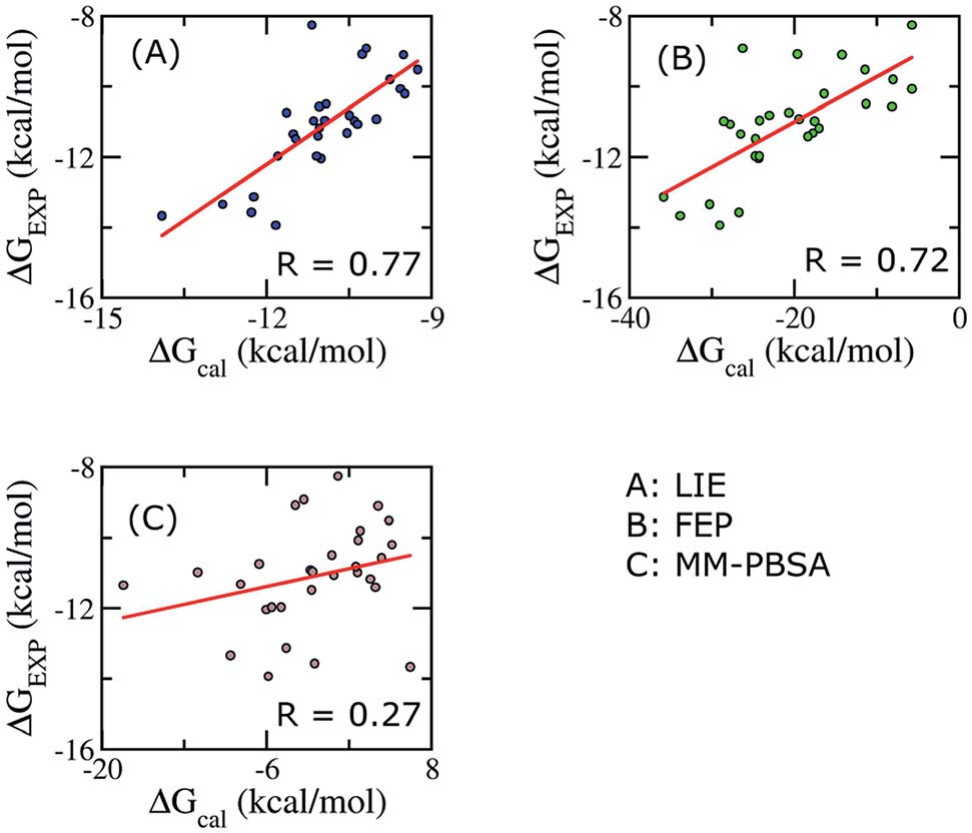
Correlations between calculated and experimental binding free energies calculated using different approaches. Three methods were applied on 30 complexes.

To establish the performance of our LIE calculation with respect to the FEP and MM-PBSA methods, the binding free energies of all ligands interacting with Aβ_11–40_ using the three methods are listed in Table S3.† The correlations between the calculated and experimental data using the three approaches are shown in Fig. 4. It should be emphasized that our groups have been successfully applied FEP and MM/PBSA to calculated binding free energy for various ligand–Aβ peptides complexes.^64,66,91,94,95^

When all of ligands are considered, the time-consuming FEP method, expected to give accurate binding affinity of protein–ligand complex, generates results with a correlation to experimental data (*R* = 0.72) similar to the LIE method with our parameter set (*R* = 0.79). In contrast, the binding affinity calculated using MM/PBSA method gives a poor correlation with experiment data (*R* = 0.27). In Aβ peptide systems, as there is no unique binding pose, the choice of the most appropriate dielectric constant value for accurate electrostatic calculations is problematic. The dependence of the MM/PBSA results on the dielectric constant values has already been discussed out on other systems; however, no systematic approach to improve the results was reported.^41,59^ A poor correlation can also come from the entropy estimation. In order to reduce the computational time, we only chose the last structures of MD simulation to calculate the entropy. Further studies are required to gain deeper insights and improve the results of MM/PBSA approach.

### CPU time consumption

The required computing resource for ligand affinity prediction to 12Aβ_11–40_ peptide using LIE approach is much less than that of MM/PBSA and/or FEP protocols. The ratio is up to *ca.* 50 times. Indeed, the MM/PBSA approach requires a huge of CPU time for estimating Δ*G*_PB_ and −*T*Δ*S* values due to the 12Aβ_40_–ligand systems consist of more than 5700 atoms. It takes more than 5700 node-hours (>8 months) to estimate the ligand-affinity for a single complexed system using MM/PBSA approach. In particular, it costs *ca.* 1200 computing node-hours for estimating Δ*G*_PB_ value over 150 snapshots of each complex. In another hand, the entropic approximation is required more computational costs, although the normal model calculation is very approximate. The entropic evaluation is serial calculation, whereas the simulated time is up to several weeks for every normal model estimation. However, it is noted that the computational time was not included the MD production time, which is of *ca.* 3.6 node-days for three independent trajectories of a complex. That also is total time to predict the binding affinity of a ligand to 12Aβ_11–40_ peptide using LIE approach, because non-covalent bond interaction energy between two molecules was recorded over simulation time intervals. Furthermore, the FEP results were obtained throughout the alchemical process, the CPU time consumption is extremely huge that it is slightly larger than MM/PBSA approach in this case.

## Conclusions

In this study, a LIE model has been successfully optimized to predict the binding affinity of ligands to 12Aβ_11–40_ protofibril showing good correlation between calculated and experimental values (*R* = 0.79) with a small error (*δ* = 0.95). We found that the standard LIE parameters cannot provide a good correlation with experimental data, which is likely due to the lack of a unique binding pose for the 12Aβ_11–40_ oligomer.

The van der Waals interactions are dominant and much more important than electrostatic interaction, which is consistent with the small number of H-bonds found in the complexes of the ligands and 12Aβ_11–40_ peptide during MD simulations. The magnitude of electrostatic interactions of the ligand in the free state is larger than that in the bound state in most of the cases, which indicates small desolvation effects of ligands.

Binding free energies of all complexes are calculated by using the FEP and the MM/PBSA methods. Compared to our LIE model, the results from the FEP approach do not provide better correlation (*R* = 0.72) with experimental data, while the MM/PBSA method provides very low correlation (*R* = 0.27).

An accurate and fast method for computing binding affinities of molecules with Aβ peptides is of great interest. Our LIE model, much less time-consuming than both FEP and MM/PBSA methods, opens the door to accurate and rapid affinity prediction of ligands with Aβ peptides and may help design new ligands.

## Conflicts of interest

There are no conflicts to declare.

## Supplementary Material

RA-009-C9RA01177C-s001
