# Supplementary material for: Adequate prediction for inhibitor affinity of Aβ40 protofibril using the linear interaction energy method
Source: RSC Adv. 2019 Apr 23;9(22):12455–61. doi: 10.1039/c9ra01177c (PMC9063661; doi:10.1039/c9ra01177c)
Supplement: RA-009-C9RA01177C-s001 [file RA-009-C9RA01177C-s001.pdf]

Electronic Supplementary Material (ESI) for RSC Advances.
This journal is © The Royal Society of Chemistry 2019

Supporting Information

Adequate Prediction for Inhibitor Affinity of 12Aβ_{40} Photofibril Using Linear Interaction Energy Method

Son Tung Ngo,[a,b,*] Binh Khanh Mai,[c,†,*] Philippe Derreumaux[def] and Van V. Vu[g]

[a]Laboratory of Theoretical and Computational Biophysics, Ton Duc Thang University, Ho Chi Minh City, Vietnam

[b]Faculty of Applied Sciences, Ton Duc Thang University, Ho Chi Minh City, Vietnam

[c]Institute for Computational Science and Technology (ICST), Quang Trung Software City, Ho Chi Minh City, Vietnam

[d]Laboratoire de Biochimie Theorique, UPR 9080 CNRS, IBPC, Universite Paris 7, 13 rue Pierre et Marie Curie, 75005, Paris, France

[e]Laboratory of Theoretical Chemistry, Ton Duc Thang University, Ho Chi Minh City, Vietnam

[f]Faculty of Pharmacy, Ton Duc Thang University, Ho Chi Minh City, Vietnam

[g]NTT Hi-Tech Institute, Nguyen Tat Thanh University, Ho Chi Minh City, Vietnam

[†]Present address: Department of Organic Chemistry, Stockholm University, SE-106 91 Stockholm, Sweden.

*Emails: ngosontung@tdtu.edu.vn and binh.khanh.mai@su.se

Table S1. List of amyloid inhibitors with PubChem ID and experimental K_i values.

N^0	Compound	Pubchem ID	Structure	K_i (nM)
1	CHEMBL81260[1]	10064445	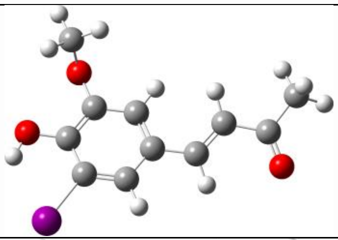	19.97
2	CHEMBL78012[2]	10133297	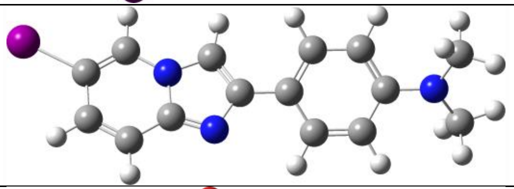	15.00
3	CHEMBL74704[3]	10184360	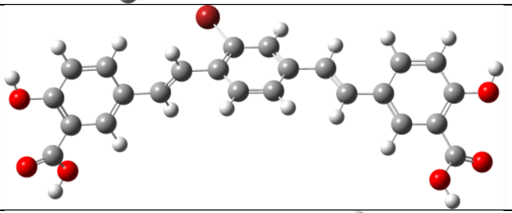	0.11
4	CHEMBL92852[4]	10335228	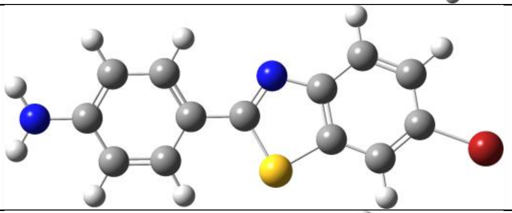	7.20
5	CHEMBL330529[4]	10422457	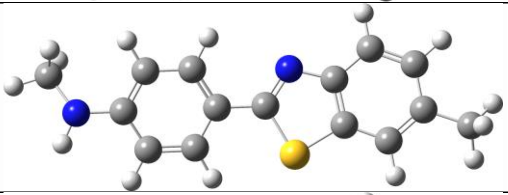	10.00
6	CHEMBL328660[4]	10426074	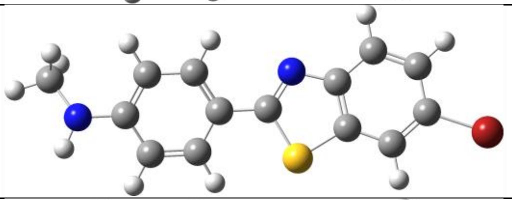	1.70
7	CHEMBL93884[4]	10444413	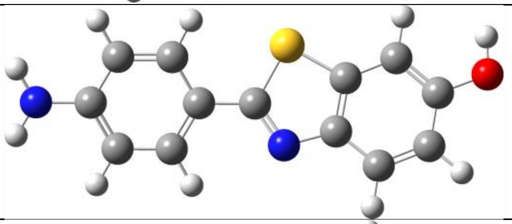	46.00
8	CHEMBL55401[5]	10496851	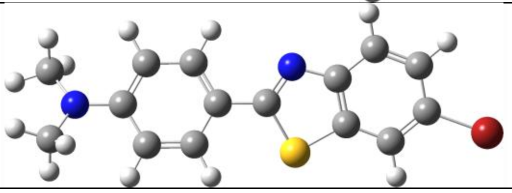	1.90

9	CHEMBL58092[5]	10844298	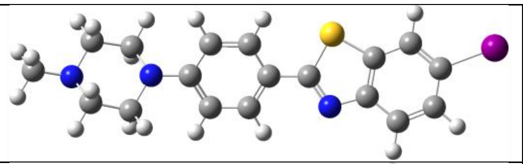	5.40
10	CHEMBL78621[3]	11054105	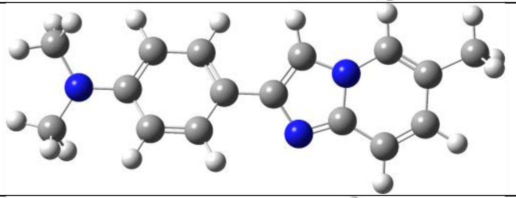	242.00
11	CHEMBL328430[4]	11129817	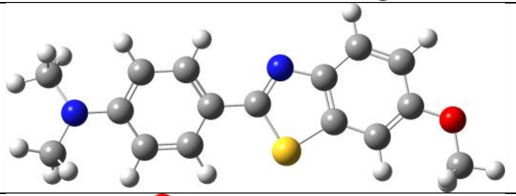	1.90
12	CHEMBL375934[6]	11574659	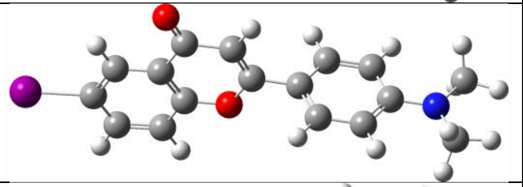	13.20
13	CHEMBL224643[6]	11632242	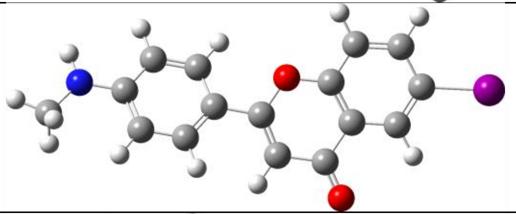	22.60
14	CHEMBL388844[6]	11703303	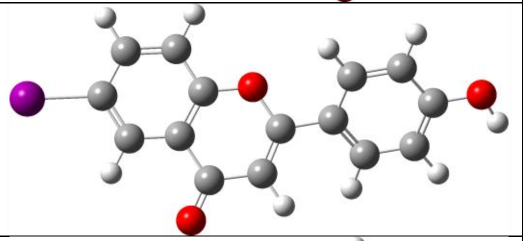	72.50
15	CHEMBL92802[4]	11821524	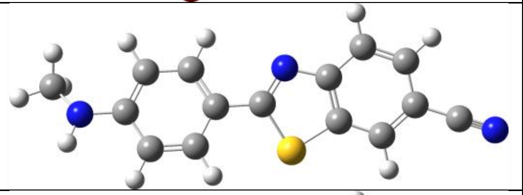	8.60
16	CHEMBL1908959[7]	1358096	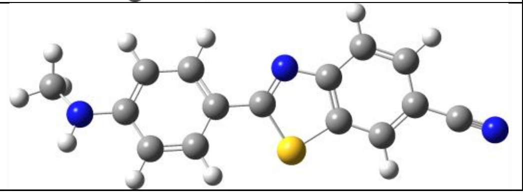	10.09

17	CHEMBL305634[2]	15521136	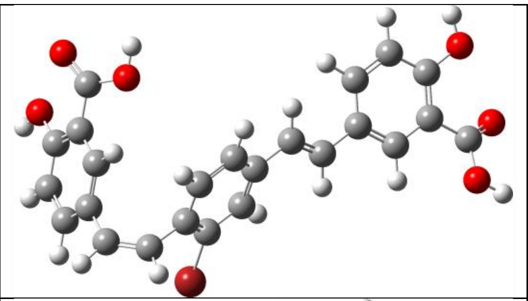	0.19
18	CHEMBL76112[2]	15521135	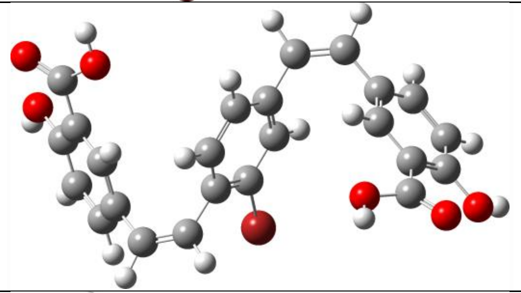	0.13
19	CHEMBL74348[2]	15521137	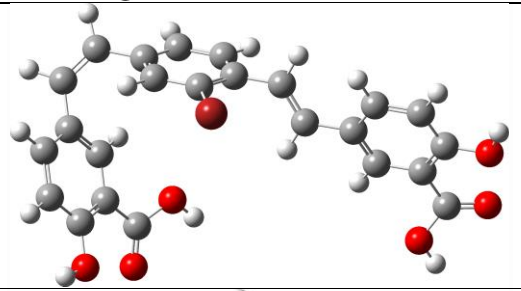	0.27
20	NAPROXEN[8]	156391	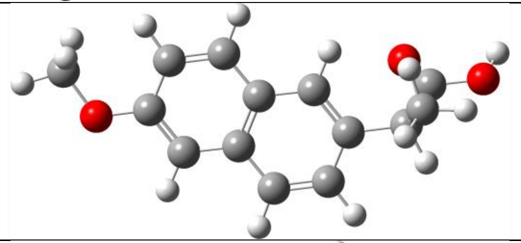	
21	(3Z,5E)-6-[4-(3-fluoropropoxy)-3-methoxyphenyl]-4-hydroxyhexa-3,5-dien-2-one[1]	16087292	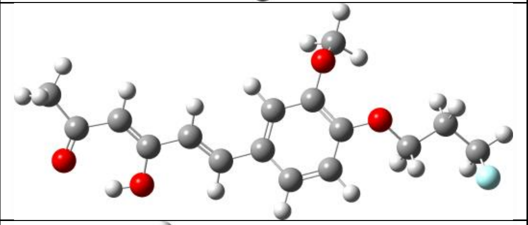	316.30
22	CHEMBL387276[1]	16087303	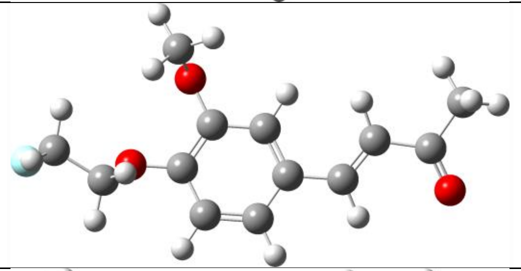	232.70
23	(3Z,5E)-6-[4-(2-fluoroethoxy)-3-methoxyphenyl]-4-hydroxyhexa-3,5-dien-2-one[1]	16087304	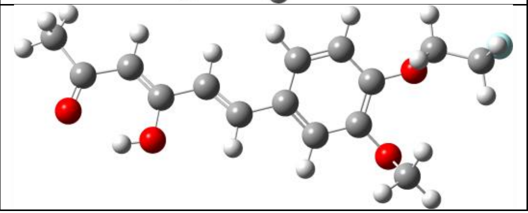	958.10

24	(1E,4Z,6E)-7-[4-(3-fluoropropoxy)-3-methoxyphenyl]-5-hydroxy-1-(4-hydroxy-3-methoxyphenyl)hepta-1,4,6-trien-3-one[1]	16087306	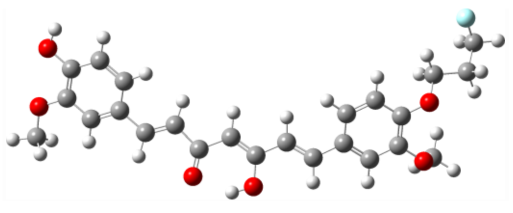	0.07
25	Thioflavin T[5]	16954	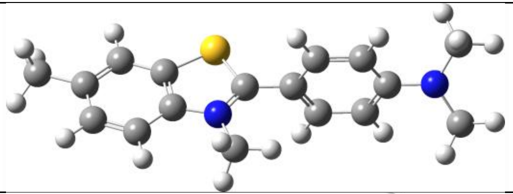	116.00
26	4-(1,3-benzothiazol-2-yl)aniline[4]	234475	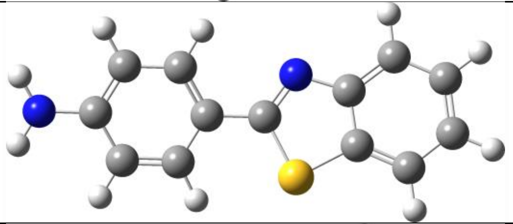	37.00
27	Pittsburgh Compound-B[9]	N/A	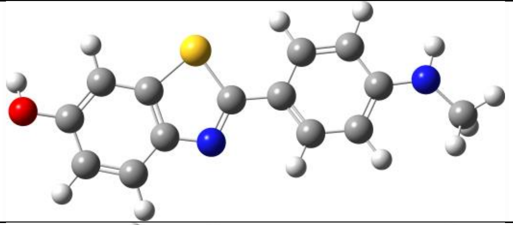	4.3
28	CHEMBL94230[4]	9835167	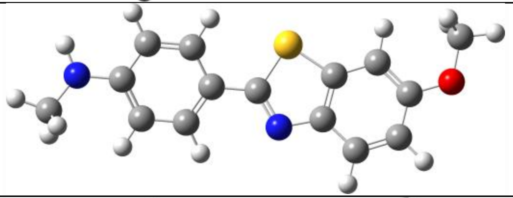	4.90
29	CHEMBL93334[4]	9837643	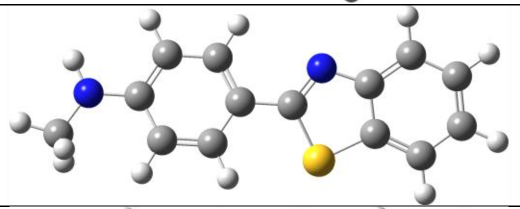	11.00
30	CHEMBL81260[3]	9839907	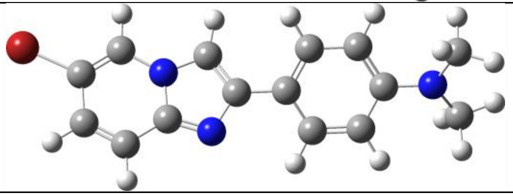	10.30

Table S2. Average interaction energies (kcal/mol) of each ligand with their surroundings at the bound and free states along their calculated LIE and experimental binding free energies.

Pubchem ID	$\langle V_{l-s}^{vdW} \rangle_b$	$\langle V_{l-s}^{vdW} \rangle_f$	$\langle V_{l-s}^{elec} \rangle_b$	$\langle V_{l-s}^{elec} \rangle_f$	ΔG_{exp}
10064445	-34.0	-17.4	-16.2	-23.7	-10.6
10133297	-42.6	-23.4	-11.1	-15.4	-10.7
10184360	-42.2	-19.6	-114.1	-144.9	-13.7
10335228	-34.5	-18.8	-10.3	-23.3	-11.2
10422457	-37.0	-22.1	-5.9	-10.3	-11.0
10426074	-39.9	-22.8	-6.2	-10.5	-12.0
10444413	-28.1	-15.8	-27.8	-30.7	-10.1
10496851	-42.0	-24.8	-4.2	-9.2	-12.0
10844298	-48.3	-29.7	-6.7	-12.5	-11.3
11054105	-37.0	-23.0	-7.5	-14.5	-9.1
11129817	-43.2	-24.0	-4.8	-12.1	-12.0
11574659	-41.1	-25.5	-14.3	-16.3	-10.8
11632242	-39.7	-23.4	-14.6	-21.2	-10.5
11703303	-32.4	-20.0	-21.3	-27.1	-9.8
11821524	-37.9	-23.4	-11.4	-16.9	-11.1
1358096	-43.7	-26.5	-6.0	-12.1	-11.0
15521136	-38.3	-18.0	-124.4	-146.1	-13.3
15521135	-37.0	-17.9	-121.0	-139.2	-13.6
15521137	-37.2	-18.0	-121.6	-138.4	-13.1
156391 (Naproxen)	-24.2	-10.9	-70.8	-87.8	-11.3
16087292	-37.7	-24.4	-12.8	-22.2	-8.9
16087303	-30.3	-19.1	-14.1	-22.1	-9.1
16087304	-38.7	-22.2	-9.7	-20.9	-8.3
16087306	-49.9	-33.1	-11.4	-34.1	-13.9
16954 (Thioflavin T)	-37.7	-25.5	-10.1	-7.2	-9.5
234475	-27.9	-15.9	-21.9	-24.5	-10.2
Pittsburgh Compound-B	-38.4	-19.6	-18.0	-21.7	-11.5
9835167	-39.8	-22.7	-9.6	-14.6	-11.4
9837643	-34.0	-20.5	-6.4	-11.5	-10.9
9839907	-40.0	-23.6	-6.6	-13.8	-11.0

Table S3. Binding free energies of each ligand with $12A\beta_{11-40}$ oligomer by using MM/PBSA and FEP methods.

Pubchem ID	ΔE_{vdW}	ΔE_{Elec}	ΔG_{sur}	ΔG_{PB}	$-T\Delta S$	$\Delta G_{MM/PBSA}$	ΔG_{FEP}
10064445	1.55	-38.67	-4.58	18.51	26.95	3.76	-8.16
10133297	-4.54	-37.20	-4.88	12.83	27.13	-6.65	-20.65
10184360	312.96	-45.13	-7.58	-283.12	29.04	6.18	-33.85
10335228	-3.00	-37.59	-4.80	21.95	26.23	2.79	-16.99
10422457	-2.42	-37.71	-5.14	20.49	26.51	1.72	-17.54
10426074	-7.37	-43.16	-4.95	22.49	26.94	-6.05	-24.34
10444413	-11.90	-33.58	-4.39	24.71	26.92	1.77	-5.75
10496851	-3.80	-44.44	-5.27	20.97	26.97	-5.57	-24.71
10844298	-4.29	-52.35	-6.34	16.81	27.98	-18.18	-26.50
11054105	-4.62	-41.57	-5.44	20.81	27.25	-3.56	-19.63
11129817	-4.56	-42.93	-5.46	20.97	27.22	-4.76	-24.24
11574659	-3.81	-43.55	-5.69	27.06	27.55	1.57	-23.04
11632242	-2.77	-40.57	-5.33	21.15	27.05	-0.47	-11.30
11703303	-9.25	-31.67	-5.16	21.53	26.49	1.94	-8.02
11821524	-3.57	-40.56	-5.31	22.24	26.91	-0.29	-27.77
1358096	-3.80	-45.01	-5.51	15.31	27.13	-11.87	-28.56
15521136	286.71	-37.26	-5.82	-281.41	28.70	-9.08	-30.28
15521135	334.64	-36.85	-6.22	-322.03	28.54	-1.92	-26.71
15521137	289.18	-36.43	-6.79	-278.45	28.17	-4.33	-35.85
156391 (Naproxen)	130.35	-35.23	-4.54	-126.29	27.50	-8.21	-17.73
16087292	-6.52	-40.37	-6.20	22.22	28.05	-2.84	-26.27
16087303	-13.53	-36.35	-5.22	31.31	27.23	3.45	-14.23
16087304	-4.31	-40.20	-5.74	21.72	28.58	0.05	-5.75
16087306	-20.55	-57.46	-7.96	49.47	30.65	-5.85	-29.05
16954 (Thioflavin T)	-216.02	-31.36	-4.71	229.23	27.24	4.38	-11.41
234475	-15.03	-29.18	-4.41	27.54	25.73	4.65	-16.37
Pittsburgh Compound-B	-9.85	-40.89	-4.72	26.48	26.81	-2.18	-24.69
9835167	-11.15	-37.99	-5.04	30.55	26.85	3.22	-18.35
9837643	-6.67	-34.73	-4.51	17.17	26.45	-2.29	-19.43
9839907	-5.46	-44.66	-5.26	26.10	27.16	-2.11	-24.21

Table S4. The binding free energies was estimated using LIE and experimental approaches of training set comprising of 20 complexes. In particular, the parameter α, β, and γ were assigned as shown as equation (2) of the main text. The unit is of kcal/mol.

Pubchem ID	ΔG_{LIE}	ΔG_{exp}
10064445	-11.05	-10.57
10184360	-13.91	-13.67
10335228	-11.04	-11.18
10426074	-11.01	-12.04
10444413	-9.57	-10.07
10844298	-11.52	-11.35
11054105	-10.26	-9.08
11574659	-10.49	-10.82
11632242	-10.92	-10.49
11703303	-9.75	-9.80
1358096	-11.14	-10.98
15521136	-12.80	-13.34
15521135	-12.28	-13.57
156391	-10.54	-11.32
16087303	-9.51	-9.10
16087304	-11.17	-8.26
16954	-9.25	-9.52
234475	-9.49	-10.20
9835167	-11.06	-11.41
9839907	-10.95	-10.96

Table S5. The binding free energies was estimated using LIE and experimental approaches of testing set comprising of 10 complexes. In particular, the parameter α, β, and γ were assigned as shown as equation (2) of the main texts. The unit is of kcal/mol.

Pubchem ID	ΔG_{LIE}	ΔG_{exp}
10133297	-11.64	-10.74
10496851	-11.09	-11.97
11821524	-10.35	-11.07
15521137	-12.24	-13.13
16087306	-11.84	-13.94
2-phenylbenzothiazoles	-11.47	-11.48
10422457	-10.40	-10.98
16087292	-10.19	-8.92
9837643	-10.01	-10.92
11129817	-11.80	-11.97

Figure S1. RMSD values during MD simulations.

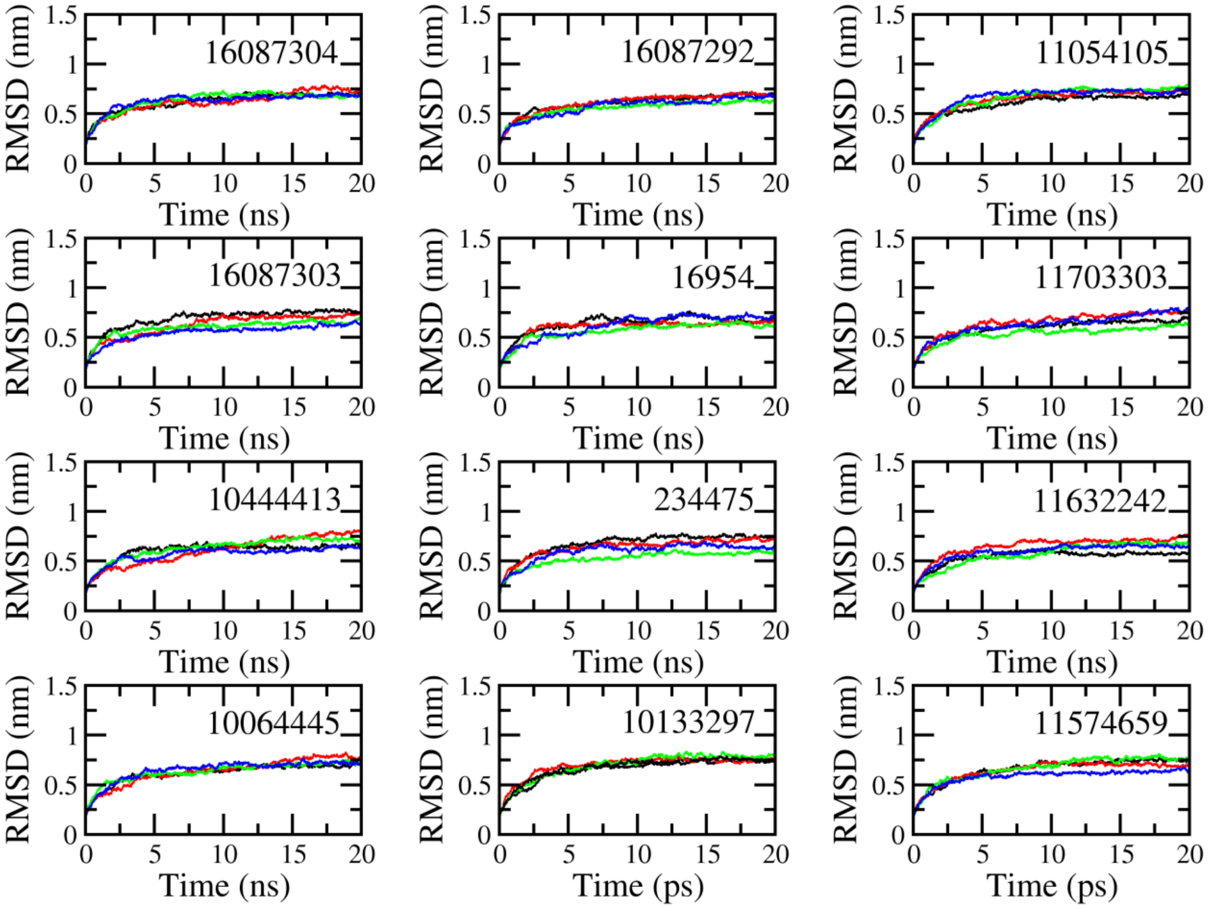

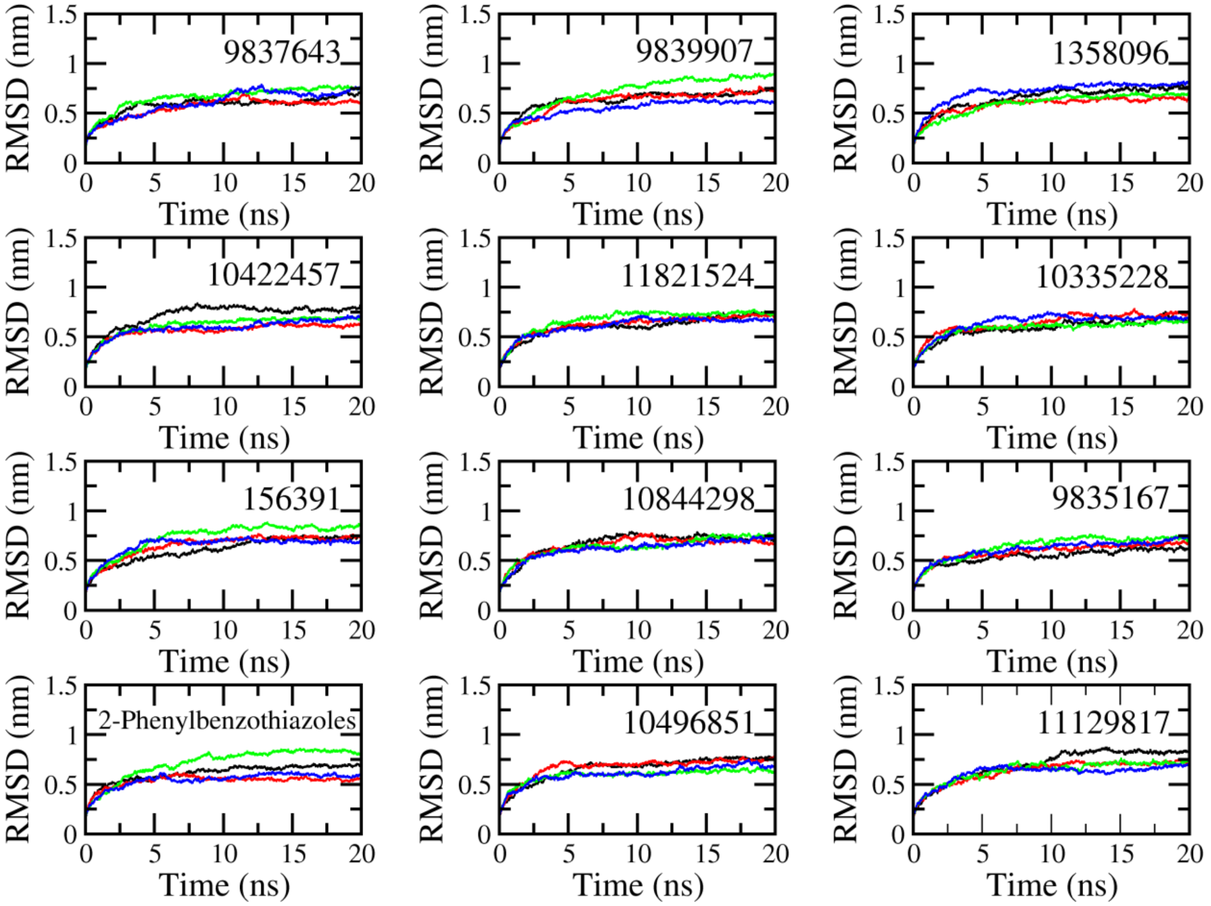
9837643
9839907
1358096
10422457
11821524
10335228
156391
10844298
9835167
2-Phenylbenzothiazoles
10496851
11129817
RMSD (nm)
Time (ns)

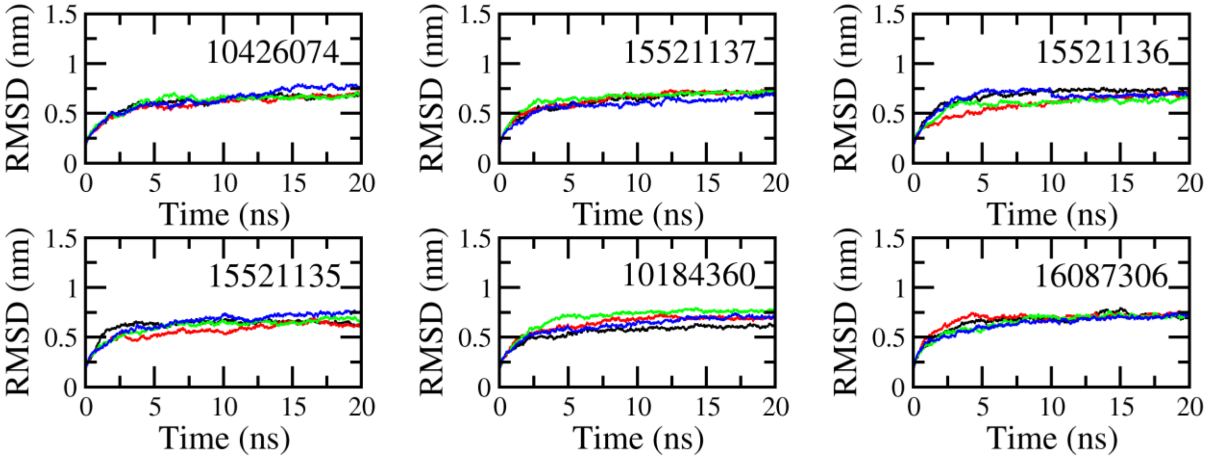
10426074
15521137
15521136
15521135
10184360
16087306
RMSD (nm)
Time (ns)

Figure S2. 2-D protein-ligand interaction diagrams

Pittsburgh Compound-B

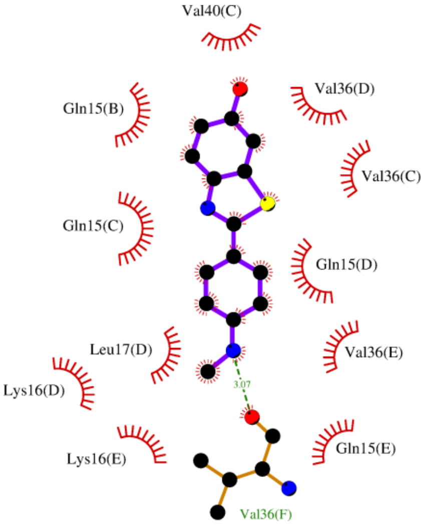

16954

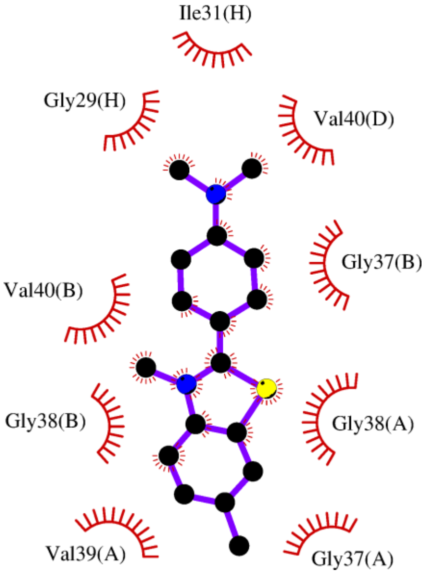

156391

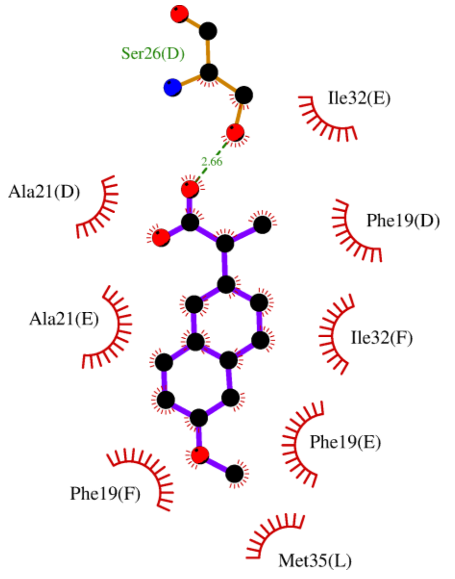

234475

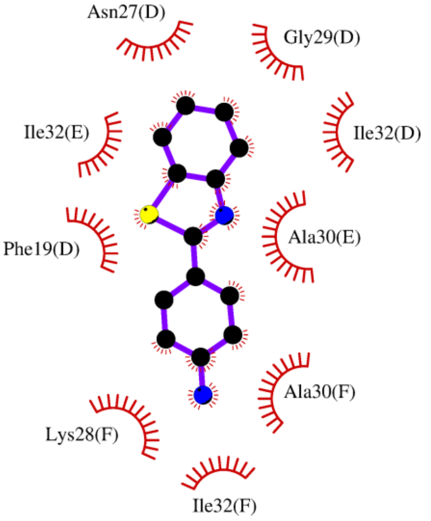

1358096

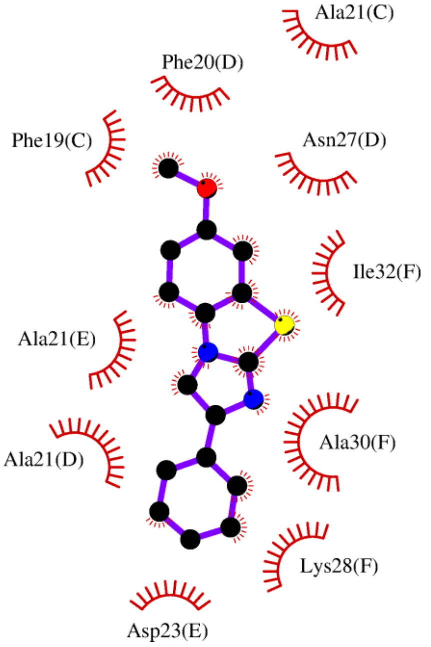

9835167

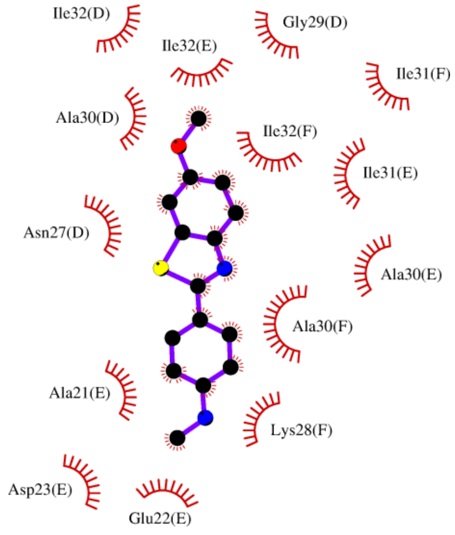

9837643

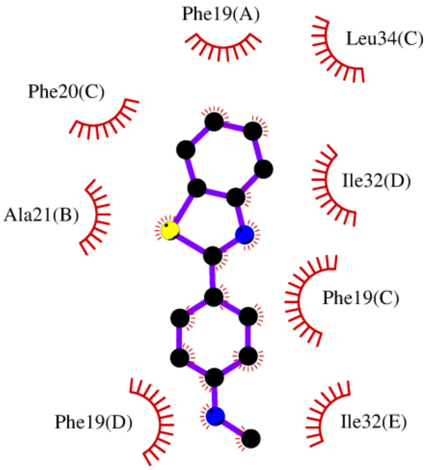

9839907

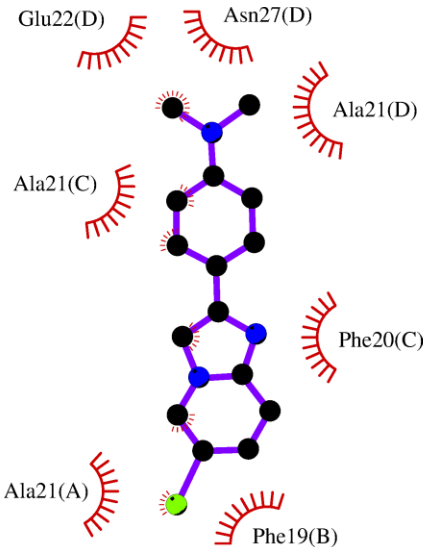

10064445

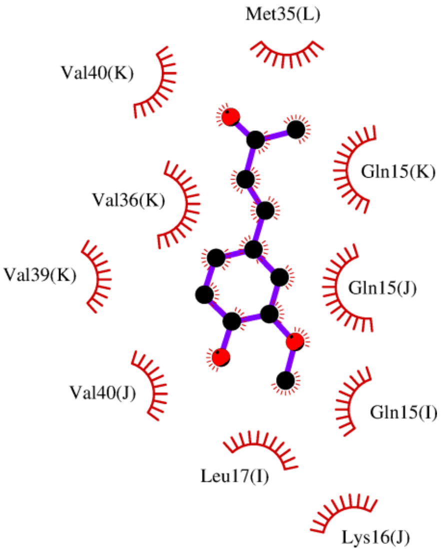

10133297

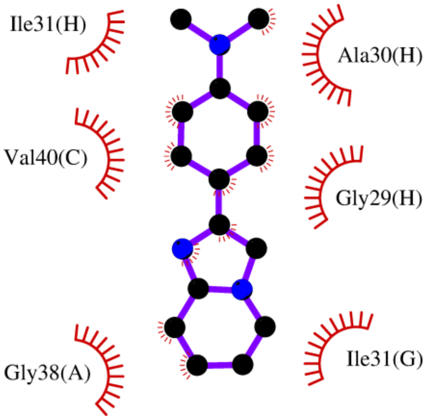

10184360

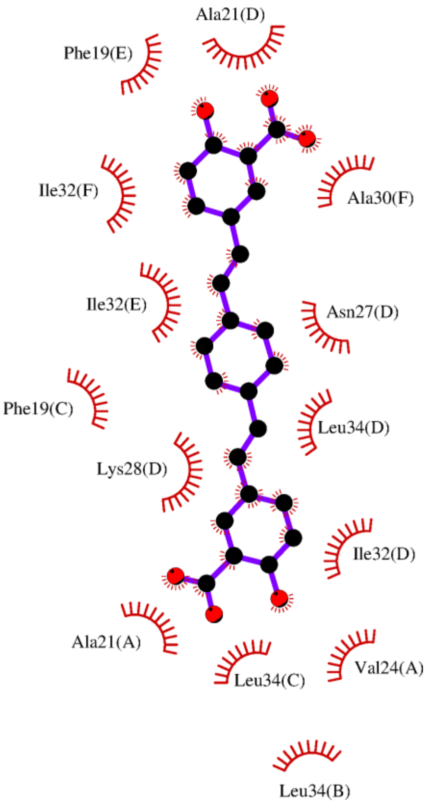

10335228

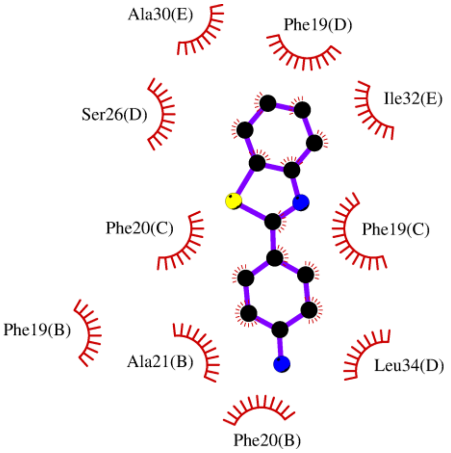

10422457

10426074

10444413

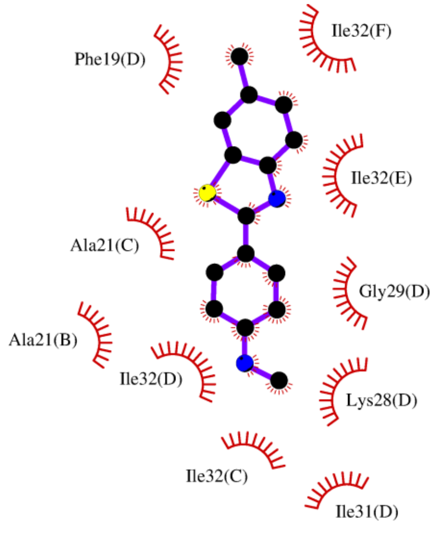

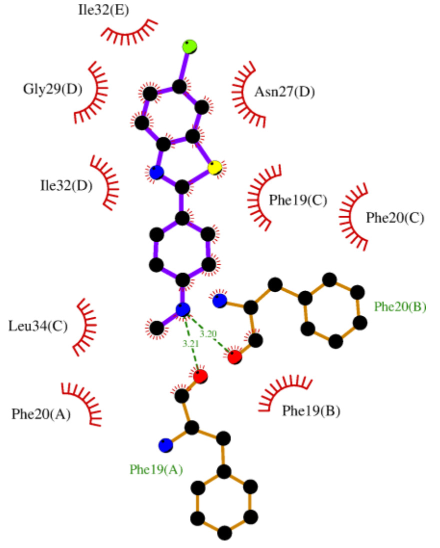

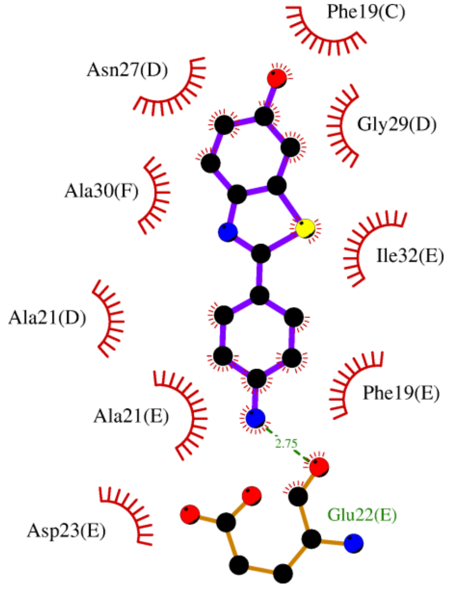

10844298

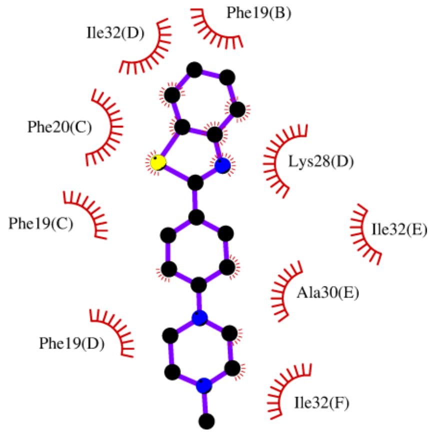

11054105

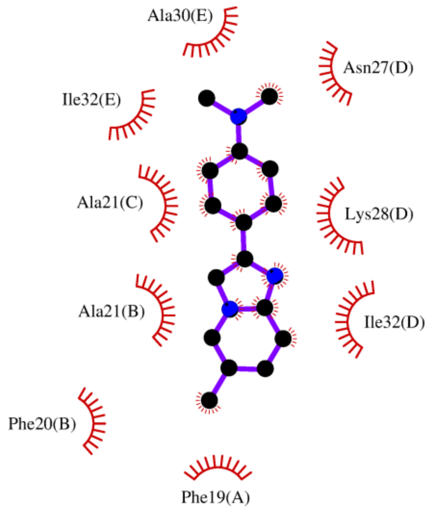

16087303

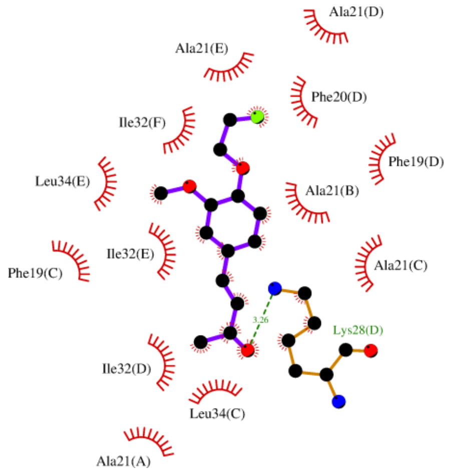

Figure S3. Number of HBs during MD simulations.

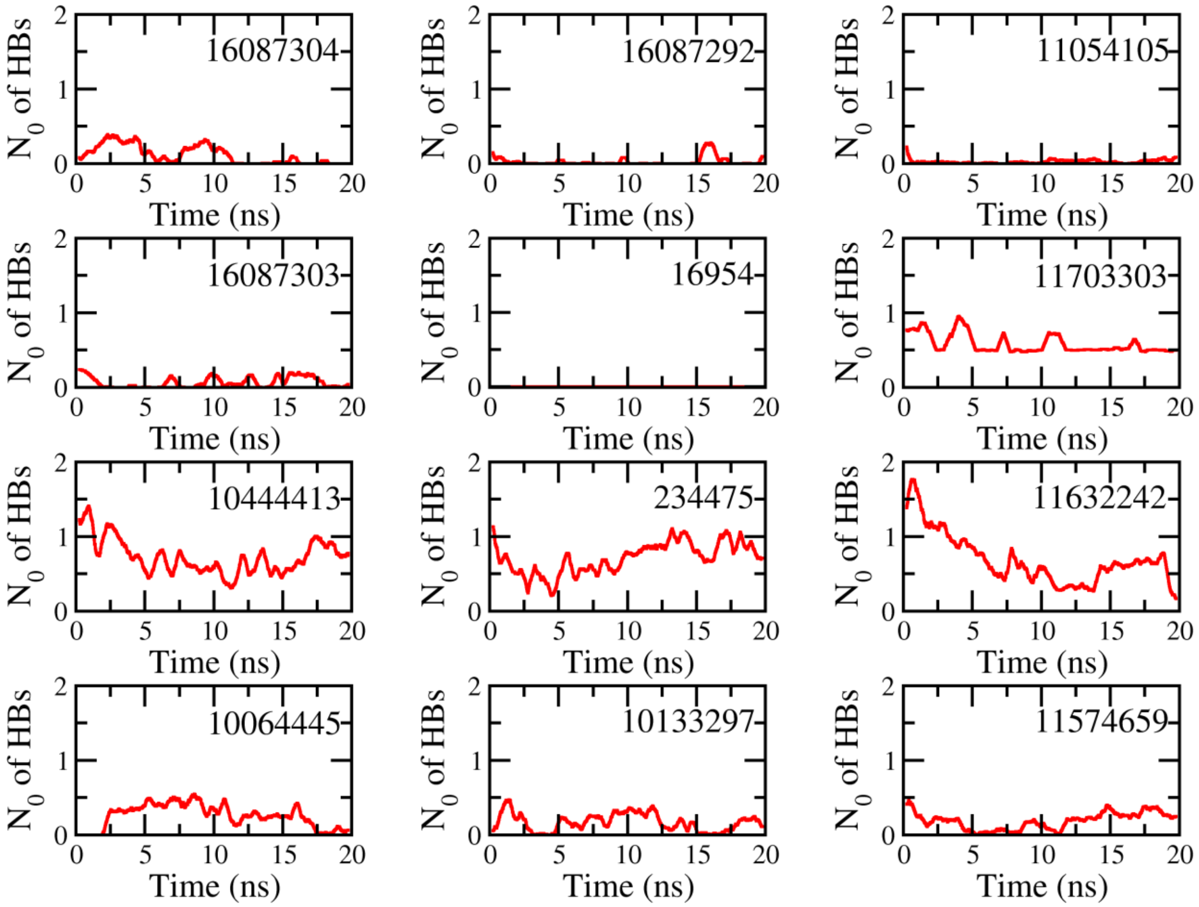

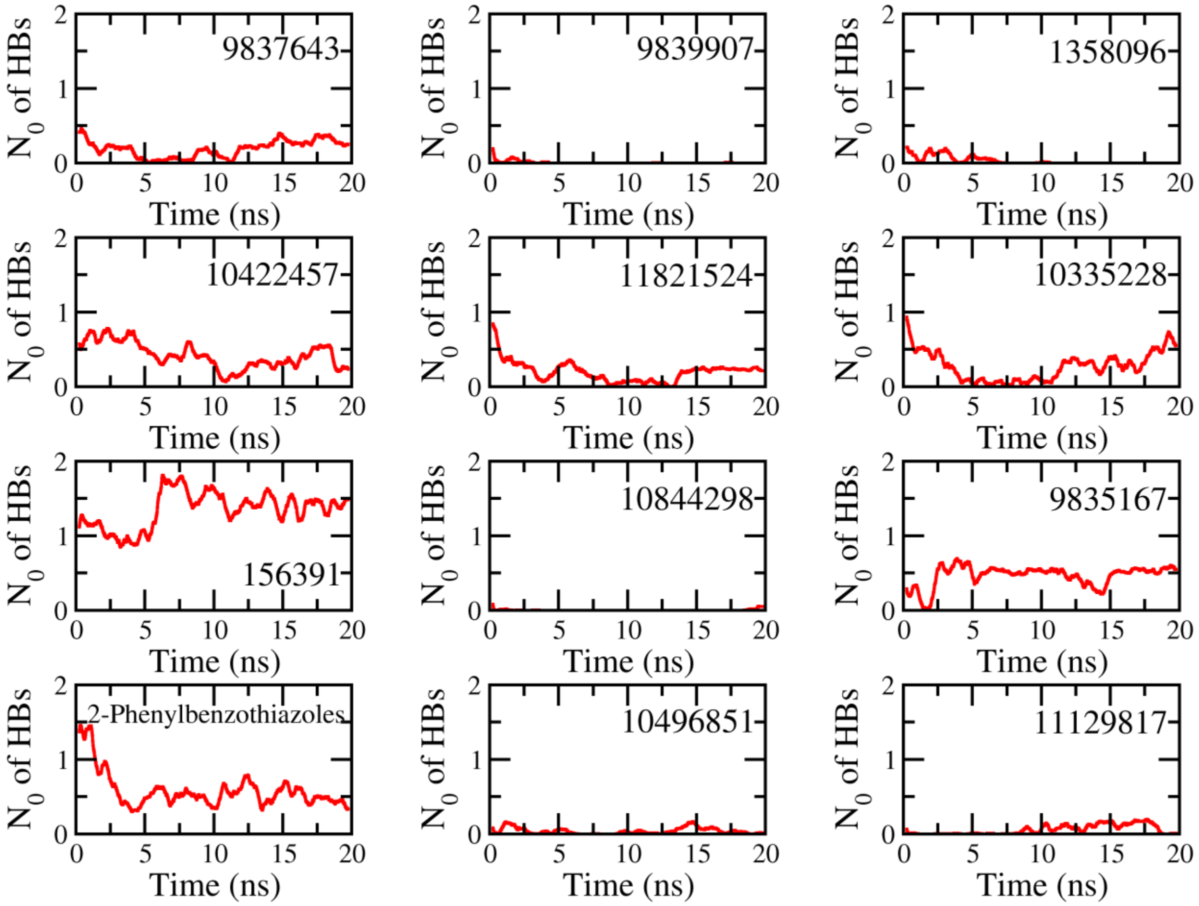
9837643
9839907
1358096
10422457
11821524
10335228
156391
10844298
9835167
2-Phenylbenzothiazoles
10496851
11129817
N_0 of HBs
Time (ns)

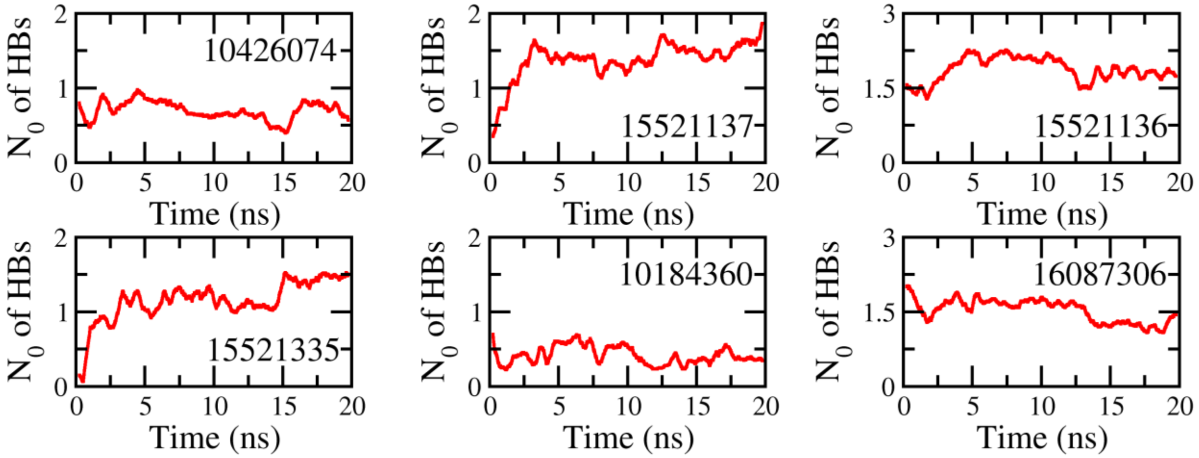
10426074
15521137
15521136
15521335
10184360
16087306
N_0 of HBs
Time (ns)

Figure S4. Correlation between experimental binding free energies and that calculated using LIE model with the separation of *β* value (eq. 3)

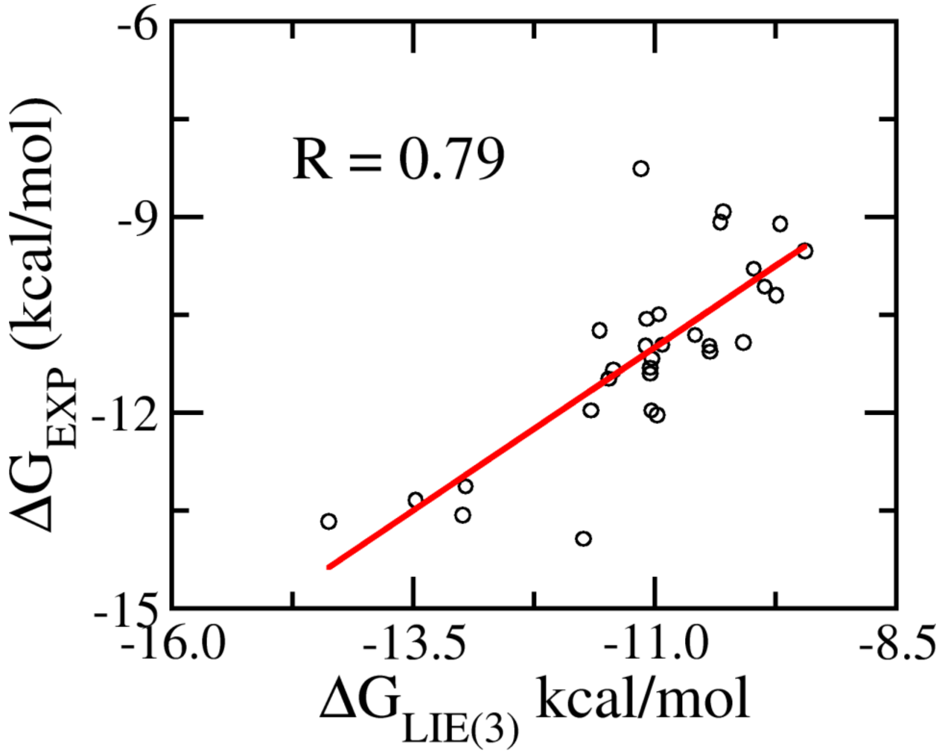

References

1. E. K. Ryu, Y. S. Choe, K. H. Lee, Y. Choi and B. T. Kim, *J. Med. Chem.*, 2006, **49**, 6111-6119.
2. C.-W. Lee, Z.-P. Zhuang, M.-P. Kung, K. Plössl, D. Skovronsky, T. Gur, C. Hou, J. Q. Trojanowski, V. M. Y. Lee and H. F. Kung, *J Med Chem*, 2001, **44**, 2270-2275.
3. Z. P. Zhuang, M. P. Kung, A. Wilson, C. W. Lee, K. Plossl, C. Hou, D. M. Holtzman and H. F. Kung, *J. Med. Chem.*, 2003, **46**, 237-243.
4. C. A. Mathis, Y. M. Wang, D. P. Holt, G. F. Huang, M. L. Debnath and W. E. Klunk, *J Med Chem*, 2003, **46**, 2740-2754.
5. Z. P. Zhuang, M. P. Kung, C. Hou, D. M. Skovronsky, T. L. Gur, K. Plossl, J. Q. Trojanowski, V. M. Y. Lee and H. F. Kung, *J. Med. Chem.*, 2001, **44**, 1905-1914.
6. M. Ono, N. Yoshida, K. Ishibashi, M. Haratake, Y. Arano, H. Mori and M. Nakayama, *J. Med. Chem.*, 2005, **48**, 7253-7260.
7. D. Alagille, H. DaCosta, R. M. Baldwin and G. D. Tamagnan, *Bioorganic Med. Chem. Lett.*, 2011, **21**, 2966-2968.
8. E. D. Agdeppa, V. Kepe, A. Petric, N. Satyamurthy, J. Liu, S. C. Huang, G. W. Small, G. M. Coled and J. R. Barrio, *Neuroscience*, 2003, **117**, 723-730.
9. K.-S. Lin, M. L. Debnath, C. A. Mathis and W. E. Klunk, *Bioorganic & Medicinal Chemistry Letters*, 2009, **19**, 2258-2262.
